# Microencapsulated Chitosan-Based Nanocapsules: A New Platform for Pulmonary Gene Delivery

**DOI:** 10.3390/pharmaceutics13091377

**Published:** 2021-08-31

**Authors:** Estefanía Fernández-Paz, Lucía Feijoo-Siota, Maria Manuela Gaspar, Noemi Csaba, Carmen Remuñán-López

**Affiliations:** 1 Nanobiofar Group, Department of Pharmacology, Pharmacy & Pharmaceutical Technology, Faculty of Pharmacy, University of Santiago de Compostela, 15782 Santiago de Compostela, Spain; estefania.fernandez.paz@rai.usc.es; 2 Department of Microbiology & Parasitology, Faculty of Pharmacy, University of Santiago de Compostela, 15782 Santiago de Compostela, Spain; feijoosiota@gmail.com; 3 Research Institute for Medicines (iMed.ULisboa), Faculty of Pharmacy, University of Lisboa, Av. Professor GamaPinto, 1649-003 Lisbon, Portugal; manuelagaspar@campus.ul.pt; 4 Nanobiofar Group, Center of Research in Molecular Medicine and Chronic Diseases (CiMUS), University of Santiago de Compostela, Campus Vida, 15706 Santiago de Compostela, Spain; noemi.csaba@usc.es

**Keywords:** chitosan nanocapsules, in vivo study, microspheres, pCMV-βGal, pulmonary gene delivery, spray-drying

## Abstract

In this work, we propose chitosan (CS)-based nanocapsules (NCs) for pulmonary gene delivery. Hyaluronic acid (HA) was incorporated in the NCs composition (HA/CS NCs) aiming to promote gene transfection in the lung epithelium. NCs were loaded with a model plasmid (pCMV-βGal) to easily evaluate their transfection capacity. The plasmid encapsulation efficiencies were of approx. 90%. To facilitate their administration to the lungs, the plasmid-loaded NCs were microencapsulated in mannitol (Ma) microspheres (MS) using a simple spray-drying technique, obtaining dry powders of adequate properties. In vivo, the MS reached the deep lung, where the plasmid-loaded CS-based NCs were released and transfected the alveolar cells more homogeneously than the control formulation of plasmid directly microencapsulated in Ma MS. The HA-containing formulation achieved the highest transfection efficiency, in a more extended area and more homogeneously distributed than the rest of tested formulations. The new micro-nanostructured platform proposed in this work represents an efficient strategy for the delivery of genetic material to the lung, with great potential for the treatment of genetic lung diseases.

## 1. Introduction

Gene therapy offers hope of treatment for rare and other serious diseases that currently have no established effective treatments. It has become a valuable strategy for the treatment of congenital or acquired genetic lung diseases, such as α_1_-antitrypsin deficiency, cystic fibrosis (CF), acute respiratory distress syndrome (ARDS), pulmonary inflammation, surfactant deficiency, hereditary pulmonary arterial hypertension and some types of lung cancer [1,2,3,4]. In addition, gene therapy is interesting to treat infective pulmonary diseases—which are caused by pathogenic bacteria, such as tuberculosis—whose progression depends on the host’s defense system and of the expression of specific bacterial genes. Skvortsov et al. described in 2013 the transcriptome of Mycobacterium tuberculosis in infected mouse lungs and identified those genes whose expression were increased with the progression of the infection [5]. These virulence genes are a good target for applying gene therapy, by the administration of siRNAs capable of interfering in their expression. Parallel to the identification of the mechanisms involved in diseases susceptible to be treated with gene therapy, great efforts are being directed to the development of efficient nanovectors that release intracellularly the genetic material.

Non-viral gene therapy vectors constitute an interesting alternative compared to viral vectors—initially proposed in gene therapy—because they are easily produced and do not present immunogenicity or other safety problems. More specifically, positively charged biopolymers are increasingly used for their preparation and, among them, chitosan (CS) stands out for its interesting features [6,7,8,9]. It is biocompatible, biodegradable (e.g., by lysozyme), mucoadhesive and it enhances the absorption of therapeutic proteins across mucosal surfaces [10,11,12,13]. Inhaled CS has shown to have a cytoprotective effect against oxidative stress in excised rat lung [14] and it has demonstrated interest for the pulmonary administration of therapeutic compounds [15,16]. Plasmid/CS ionic complexes were prepared by electrostatic interactions between DNA (polyanion) and CS (polycation) [17]. The high density of positive charges existing along the CS polymeric chain (due to amino groups) was relevant in order to condense the genetic material. Further, our group proposed the entrapment of genetic material in CS nanoparticles (NPs), obtained by ionotropic gelation of CS and its counterion sodium tripolyphosphate (TPP) [18]. NPs provided protection and stability, cell uptake promotion and a great transfection capacity of the genetic material [19]. These benefits are intensified when hyaluronic acid (HA) is included in the nanocarrier composition [20]. HA is a linear, non-sulphated, biocompatible, mucoadhesive and biodegradable endogenous glycosaminoglycan [21,22]. It is found in human tissues and fluids, as well as on the surface of alveolar epithelial cells, providing protection against tissue damage in respiratory diseases [23] and preventing pleural thickening in patients with tuberculosis [24]. It has been investigated as component of drug and gene delivery systems by different routes [22,25,26], especially for pulmonary inhalation since it improves the bioavailability and transfection [26]. The incorporation of HA in CS-based nanostructures leads to gene therapy nanocarriers with enhanced synergistic properties, displaying higher biocompatibility, more efficient mucosal penetration, a better control on drug release, as well as an improved bioavailability, transfection efficiency and in vivo bioactivity [20,26,27,28,29]. HA has been reported to reduce non-specific interactions of the nanostructures with serum proteins and to increase their internalization in cells that express CD44, HARLEC or RHAMM receptors [20]. After the cell internalization, the nanocarriers containing HA are incorporated into non-lysosomal vesicular compartments and quickly are accumulated in the perinuclear area and in the cell nucleus, improving the gene expression [20]. Furthermore, it is thought that HA acts as a transcriptional activator surely by weakening of the binding between the gene material and the nanostructure [20]. It is important to highlight the good transfection results obtained in preclinical studies following the nasal and pulmonary administration of HA and CS-based NPs [20,27]. In this sense, the non-viral vectors constituted with CS and HA are promising for the delivery of genetic material (pDNA, siRNA, etc.).

CS nanocapsules (NCs)—prepared by the solvent displacement method [30] and composed by an oily core surrounded by a CS shell (core-shell structure)—have gained particular attention as drug delivery carriers due to their versatile and tunable structure that conditions their physicochemical properties [31,32,33]. The polymeric shell provides to the vector desirable pharmaceutical properties as drug protection, prolonged stability and targeting [31,34]. NCs have the capacity to load active ingredients (AIs) of low and high molecular weight (Mw), of lipophilic or hydrophilic nature, with high loading capacity [35]. They have been assayed as carriers of lipophilic molecules—like anti-inflammatory, antibacterial, and antitumoral drugs (as docetaxel, for the treatment of breast, ovarian and non-small cells lung cancer)—and hydrosoluble AIs—such as peptides, proteins, vaccines (as tetanus toxoid), antigens (as of recombinant hepatitis B surface, influenza and IutA E. coli) and polynucleotides, among many others [30,31,36]. In this sense, CS-based NCs are very interesting in gene therapy because they allow the combined administration of genetic material (hydrophilic) on their surface, as well as an adjuvant molecule (lipophilic) in the oily nucleus [37]. CS-based NCs were administered in vivo by the oral, nasal and ocular routes, but never before by the pulmonary route for gene therapy purposes [1,2,3,4], as in this study. In fact, they have recently been evaluated in vitro for gene therapy of CF—by administration of wtCFTR-mRNA in the CF cell line CFBE41o- [37]—confirming their interest in this field. However, their potential for lung gene therapy has not been investigated in vivo.

The pulmonary administration of nanostructures requires their formulation in an adequate system that allows them to overcome the barriers until reaching the desired region of the lung. For that, the advantages of the pulmonary route [38,39,40] must be taken into account, but also the possible obstacles in order to get the therapeutic goal [41]. The pulmonary via is used for either local or systemic treatments [38]. The lungs have a large surface (over 100 m^2^), an extremely small alveolar epithelium, and a great vascularization that led to a high and fast absorption [39]. On the other hand, the direct delivery of the drug in the lungs to treat respiratory diseases, such as infectious processes (e.g., tuberculosis), is very attractive because the administered dose and the side effects can be diminished, compared with other routes, like the oral [38,40]. However, in addition to the complex structure of the airways, there are several lung defense mechanisms that oppose the administration of drugs. These are the mucociliary clearance (responsible for trapping and expelling foreign particles) and the alveolar macrophages (due to their phagocytic activity), as well as a series of enzymes (lysozyme, protease inhibitors and isoenzymes of Cytochrome P-450 family) [39]. In this regard, mucoadhesive polymeric nanostructured systems have recently gained interest thanks to their ability to evade the macrophage uptake [42]. In addition, among the most promising strategies to overcome pulmonary obstacles, it is worth mentioning the design of microparticles with adequate morphology and size [43]. Mannitol (Ma) is a non-reducing sugar included in the list of Inactive Ingredients in Approved Drug Products of the Food and Drug Administration (FDA) [44]. Furthermore, according to the World Health Organization (WHO), Ma is on the Model List of Essential Medicines [45] and currently is marketed for a variety of pharmaceutical products [46]. Ma presents good spray-drying properties [47], is thermoprotective, increases the stability time of the powders with respect to other excipients [48], improves flowability and dispersibility of the powders [49] and promotes the nanosystems penetration through the mucus in the lungs due to its mucolytic and osmotic effect [50].

Taking all this into consideration and encouraged by the aforementioned advantages, the objective of this study was to develop a micro-nanoplatform for pulmonary gene therapy consisting of CS-based NCs loaded with genetic material and microencapsulated in Ma microspheres (MS). pCMV-βGal (or LacZ gene)—which encodes the enzyme β-galactosidase (β-Gal)—was used as model plasmid. Here, the preparation and characterization of the gene pulmonary delivery micro-nanoplatform is described and its potential for in vivo lung transfection in rats is evaluated.

We started from the experience and knowledge anteriorly acquired. We have previously proposed the microencapsulation of NPs of different nature in inert MS for pulmonary delivery of AIs, as proteins and antibiotics with encouraging results [13,48,51,52,53]. Specifically, CS NPs, solid lipid nanoparticles (SLN) and metal-organic frameworks (MOFs) were microencapsulated in Ma MS using a simple spray-drying technique [13,51,54], resulting in dry powders of suitable characteristics for their successful administration to the deep lung, as demonstrated in in vivo studies [13,54]. This is the first part of a larger study in which a genetic material (hydrophilic) will be combined with a lipophilic AI for the treatment of a specific genetic lung disease.

## 2. Materials and Methods

### 2.1. Chemicals

Ultrapure chitosan, hydrochloride salt (CS, Protasan^®^ UP CL 113, deacetylation degree = 75–90%, molecular weight (Mw) < 150 KDa) was purchased from Pronova Biopolymer, A.S. (Drammen, Norway); soybean lecithin (Epikuron^®^ 145V) was provided by Cargill (Madrid, Spain); Miglyol^®^ 812 N, a neutral oil composed of capric and caprylic acids (medium-chain fatty acids), was donated by Cremer Oleo Division (Hamburg, Germany); hyaluronic acid (HA, Mw ~ 166 kDa) was a present from Bioiberica (Barcelona, Spain); Curosurf^®^ (pulmonary surfactant) of Angelini Pharmacêutica (Lisbon, Portugal) was generously donated by Professor Almeida (University of Lisboa, Portugal); D-mannitol (Ma) (Mw: 182.17 g/mol), phosphate buffered saline tablets (PBS, pH = 7.4), β-Galactosidase Reporter Gene Staining kit, Nuclear Fast Red solution and Coumarin 6 (Cu^6^) (> 99%) were purchased from Sigma Aldrich (Madrid, Spain); and PureLink^TM^ HiPure Expi Plasmid DNA Gigaprep Kit was obtained from ThermoFisher Scientific (Madrid, Spain). Acetone and ethanol were grade HPLC; and ultrapure water (MilliQ, Millipore Ibérica, Madrid, Spain) was used. All other chemicals were reagent grade.

### 2.2. Production of pCMV-βGal

Competent *Escherichia coli* DH5α cells were thawed on ice and 0.11 µg of pCMV-βGal were mixed gently with *E. coli*, remaining 30 min on the ice. Then, it was incubated for 45 s in a bath at 42 °C and another 5 min on the ice. After the thermal shock, the bacteria were placed in a volume of 1 mL of Luria Bertani medium (LB) for 1 h, at 37 °C under stirring (260 rpm) and then 100 µL were grown on LB ampicillin-agar plates (100 µg/mL) and incubated during 14 h at 37 °C. One colony was incubated in 5 mL of LB medium supplemented with ampicillin for 20 h at 37 °C. After that time, the 5 mL of bacteria were transferred to a volume of 400 mL of LB (containing 400 µL of antibiotic) and allowed to incubate 24 h at 37 °C under agitation (320 rpm). After that time, the 400 mL of bacteria were taken to a volume of 3200 mL and incubated for 24 h at 37 °C. The bacteria were then collected by centrifugation and frozen until use. The purification of pCMV-βGal was performed according to the protocol specified in PureLink^TM^ HiPure Expi Plasmid DNA Gigaprep Kit (ThermoFisher Scientific, Madrid, Spain). The plasmid was checked by a 1% agarose gel containing 12 µL of SYBR Safe DNA gel stain at 70 mV and 90 min (Electrophoresis PowerPac 300, Bio Rad, Hercules, CA, USA). The concentration of the extracted plasmid was quantified by a Nanodrop 1000 Spectrophotometer (ThermoFisher Scientific, Franklin, TN, USA) and the plasmid samples were frozen at −20 °C until further studies.

### 2.3. Preparation of CS-Based NCs

The CS NCs were prepared using a solvent displacement procedure previously developed by our group in “two steps” [35], which in this work was modified and simplified to be carried out in “one step”. Briefly, an organic phase composed of 10 mg of lecithin (dissolved in 250 µL of ethanol at 37 °C), 31.2 µL of Miglyol^®^ 812N and 4.75 mL of acetone was added quickly over an aqueous phase consisting in 10 mL of a CS solution (0.25 mg/mL) under constant magnetic stirring (2000 rpm) for 10 min. Immediately, the mixture turned milky. Next, the solvents were eliminated under vacuum (Rotavapor Büchi R-210^®^, Flawil, Switzerland) to a final volume of 5 mL. Here, the CS NCs were further modified by incorporating HA to their surface. This was done by addition of HA (625 µg/mL) to the CS NCs suspension, producing HA/CS NCs. The association of the pCMV-βGal to the CS NCs (pCMV-βGal-CS NCs) was performed by its drop-by-drop addition on the previously prepared NCs suspension and incubation with gentle magnetic stirring at room temperature for 1 h. To prepare plasmid-loaded HA/CS NCs (pCMV-βGal-HA/CS NCs), the plasmid was firstly added to the CS NCs suspension and next, the HA. Specifically, the volumes and concentrations of the components employed to obtain the pCMV-βGal-loaded NCs are shown in Table 1.

### 2.4. Determination of NCs Production Yield

The NCs production yield was determined by gravimetry. For that, fixed volumes of NCs suspensions were centrifuged (60,000 rpm, 1 h, 15 °C) in an Ultracentrifuge Beckman Coulter (Optima^TM^ TLX Ultracentrifuge. Rotor: TLA_100.3, Ramsey, MN, USA). The NCs creams were freeze-dried (over 24 h at −80 °C and gradual ascent until 20 °C), using a Telstar Freeze Dryer (Telstar LyoQuest −85, Barcelona, Spain) (*n* = 3).

The production yield (P.Y.) was calculated using the following formula:

(1) $$P.Y.\;\left(\%\right)\;=\;\frac{\mathrm{NCs}\;\mathrm{weight}}{\mathrm{Total}\;\mathrm{solids}\;\mathrm{weight}}\times \;100$$

where total solids = CS + (HA) + Lecithin + Miglyol^®^

### 2.5. Characterization of NCs

The NCs morphology was characterized by transmission electron microscopy (TEM) (Jem-2010 Electron Microscope, Oberkochen, Germany) at 120 KV. For TEM observation, 10 µL of sample were placed on copper grids with carbon films and were stained for 2 min with 2% (*w/v*) phosphotungstic acid. Their size and ζ-potential were determined by Dynamic Light Scattering (DLS) and Laser Doppler Anemometry (LDA) using a Zetasizer^®^ Nano-ZS (Malvern Instruments, Malvern, Worcestershire, United Kingdom). The analysis was performed at 25 °C. Size and ζ-potential of each sample was measured in triplicate (*n* = 3).

### 2.6. Preliminary Study of the pCMV-βGal Association to the NCs

The association of plasmid to the CS-based NCs was determined qualitatively by agarose gel electrophoresis (1% agarose, SYBR Safe DNA gel stain, 70 mV, 90 min). The samples were prepared in 96-well plates. Free plasmid (pCMV-βGal, 5% with respect to the amount of CS used to prepare the NCs) and control NCs (without plasmid) were introduced in the first and second wells of the gel, respectively. CS-based NCs with increasing amounts of plasmid—from 10% (*w/w*) to 100% (*w/w*) with respect to the total amount of CS used to prepare the NCs—were included in the next wells. The images of the gels were obtained with a Gel Doc^TM^ XR 302 nm ultraviolet light system (Bio-Rad, Hercules, CA, USA).

### 2.7. Determination of the pCMV-βGal Association to the NCs

The association efficiency was quantitatively determined by measuring the difference between the added total plasmid amount and the free plasmid amount. For that, pCMV-βGal-CS-based NCs were centrifuged in an ultracentrifuge at 60000 rpm, during 1 h at 15 °C (Ultracentrifuge Beckman Coulter: Optima^TM^ TLX Ultracentrifuge. Rotor: TLA_100.3, Ramsey, MN, USA). The NCs creams were discarded and the liquids containing free plasmid were quantified by fluorimetry using a Qubit^®^ 3.0 Fluorometer (Invitrogen Life Technologies, Madrid, España). The experiment was done in triplicate (*n* = 3). The encapsulation efficiency (E.E.) and drug loading (D.L.) of plasmid were calculated using the following formulas:

(2) $$E.E.\;\left(\%\right)\;=\;\frac{\mathrm{Total}\;\mathrm{plasmid}\;\mathrm{amount}\;-\;\mathrm{Free}\;\mathrm{plasmid}\;\mathrm{amount}}{\mathrm{Total}\;\mathrm{plasmid}\;\mathrm{amount}}\times \;100$$

(3) $$D.L.\;\left(\%\right)\;=\;\frac{\mathrm{Total}\;\mathrm{plasmid}\;\mathrm{amount}\;-\;\mathrm{Free}\;\mathrm{plasmid}\;\mathrm{amount}}{\mathrm{CS-based}\;\mathrm{NCs}\;\mathrm{amount}}\times \;100$$

### 2.8. Preparation of Dry Powders and Determination of Spray-Drying Process Yield

NCs suspensions (CS NCs and HA/CS NCs) (volume: 4.373 mL, concentration: 34.3 mg of NCs/mL) were mixed with an appropriate volume of Ma solution (15.627 mL at 13.12%, *w/v*) to reach a NCs:Ma ratio of 1:15 (*w/w*) and a total solids content (t.s.c.) of 11% (*w/v*). For microencapsulation of pCMV-βGal-Cs-based NCs in Ma MS (pCMV-βGal-Cs-based NCs-loaded Ma MS), the t.s.c. was adjusted to 5.5% (*w/v*), while for microencapsulation of pCMV-βGal (without NCs) (pCMV-βGal-Ma MS), the t.s.c. was fixed to 5.1% (*w/v*). The mixtures were atomized using a spray-dryer (Büchi^®^ Mini Spray Dryer B-290, Flawil, Switzerland) to obtain dry powders. The employed spray-drying conditions were a feed rate of 5 mL/min, aspirator at 100%, nozzle diameter of 0.7 mm, nozzle cleaner fixed at 5, inlet temperature (T_Inlet_) was kept at 105 ± 2 °C and the air flow rate at 600 Nl/h. The powders were collected and stored at room temperature in a desiccator until use.

Spray-drying process yield (P.Y.) was determined by gravimetry. The employed total solids amount was compared with the final amount of powder obtained by spray-drying (*n* = 3) as follows:

(4) $$P.Y.\;\left(\%\right)\;=\;\frac{\mathrm{Dry}\;\mathrm{powder}\;\mathrm{weight}}{\mathrm{Total}\;\mathrm{solids}\;(\mathrm{NCs}+\mathrm{Ma})\;\mathrm{weight}\;}\times \;100$$

### 2.9. Characterization of the Dry Powders

The morphological and size characterization of the MS was performed by scanning electron microscopy (SEM) (FESEM Ultra-Plus, Zeiss, Oberkochen, Germany). The powders samples were prepared over stubs using a double-sided adhesive graphite disc and covered with a film of iridium (10 nm) employing an Emitechk 550 Sputter Coater (Manchester, United Kingdom). The sizes were determined as the distance between two tangents on opposite sides of each MS (Feret’s diameter) with the z-SmartTiff program. The average of Feret´s diameters is the geometric diameter (D_g_) and was calculated as the mean of 50 particle measurements (*n* = 50).

The apparent density of a powder (g/cm^3^), which is the mass that occupies in 1 cm^3^, was obtained using a tap density tester (Tecnociencia, A Coruña, Spain). For that, a volume of powder of weight comprised between 1.0–1.6 g was measured into a 10 mL test-tube before the mechanical tapping. The test-tube was tapped to a speed of 30 tap/min until the sample reached a constant volume [55] (*n* = 3). The real density of the dry powder samples (g/cm^3^) was determined by a helium pycnometer (Quantachrome MPY-2, New York, NY, USA) (*n* = 3).

The aerodynamic diameter (D_aer_), which is the diameter of a sphere of unit density with the same terminal settlement velocity as the particle under consideration, was theoretically calculated using the equation [56]:

(5) $$D_{\mathrm{aer}}=D_{g}\sqrt{\frac{\rho_{\mathrm{real}}}{\rho_{0}\lambda }}$$

where: D_g_: geometric diameter (µm), ρ_real_: real density of MS (g/cm^3^), ρ_0_ = 1 g/cm^3^ and λ: MS dynamic shape factor (spherical MS: λ = 1; irregular MS: λ = 2) [57].

### 2.10. Release of NCs from Dry Powders

Amounts of 100 mg of powders were incubated in 3 mL of MilliQ water or simulated pulmonary medium (0.1% Curosurf^®^ in 10 mM PBS (*v/v*, pH = 7.4)) under magnetic stirring (300 rpm, CIMAREC i Multipoint, ThermoFisher Scientific, Madrid, Spain) at room temperature and 37 °C, respectively. At different times (0, 0.5, 1, 2 and 4 h), the released NCs were analyzed to determine their size and ζ-potential using a Zetasizer, as previously described (*n* = 3). The NCs morphology was observed in samples obtained at 1 h post-release in MilliQ water by TEM, as indicated above.

### 2.11. In Vivo Studies

In vivo studies were made in accordance with the Principles of Laboratory Animal Care of the University of Santiago de Compostela, Spain, and Faculty of Pharmacy of the University of Lisbon, Portugal, and approved by the competent national authority, Direção Geral de Alimentação e Veterinária (DGAV) of Portugal in accordance with the EU Directive (2010/63/UE) for use and care of animals in research. 

Rats (female Wistar-Kyoto, 200–250 g) were provided by Charles River (Barcelona, Spain). They were aleatory separated in boxes of two animals per cage and stayed with air-conditioned at 20–24 °C with a 12 h light/dark cycle and had free access to water and food until and after the powder administration. Rats were anesthetized with a saline solution of ketamine and xylazine by inguinal injection (right groin). Anesthetized rats were positioned with the ventral side upwards, and the trachea was exposed by means of a longitudinal incision in the neck to administer the powder formulations. Samples of 20 mg of each dry powder were administered through a tracheal cannula (adaptor in Y, opening of 1.3 mm) connected to a Harvard^®^ ventilator (Inspira ASV 55-7058, 80 breaths/min, tidal volume = 1.53 cm^3^, Harvard Apparatus, Holliston, MA, USA), simulating the physiological breathing mode of rats.

#### 2.11.1. Lung Distribution of Microencapsulated NCs

For this study, the CS-based NCs were labelled with Coumarin 6 (Cu^6^) (Cu^6^-CS NCs and Cu^6^-HA/CS NCs). For that, 4.5 µL of Cu^6^ (10 mg Cu^6^/mL dichloromethane) was added and homogenized in the oil phase during the NCs preparation. Cu^6^-NCs were characterized in terms of size and ζ-potential, as described above. Prior to their microencapsulation in Ma MS, the labelled NCs were dialyzed overnight using a dialysis membrane (Spectra/Por^®^ 3 Dialysis Membrane Standard RC Tubing MWCO: 3.5 kD) to remove possible non-encapsulated Cu^6^ residues and their physicochemical properties were characterized again. To verify that there was no release of Cu^6^ from the nanosystems, the pre-dialyzed labelled NCs (with presence of non-encapsulated Cu^6^ residues) and the dialyzed labelled NCs were compared by confocal laser scanning microscopy (CLSM). The photographs were taken using a microscope Leica TCS-SP5X-AOBS with a white laser (470–670 nm) and a UV laser (Leica) with LAS AF (Leica Application Suite Advanced Fluorescence) software, using a 63X objective (oil PL APO63x/N.A.1.4-0.6 CS). Data were collected using a green channel (Cu^6^ emission, λ = 455–461 nm).

An amount of 20 mg of dry powder (including 1.4 mg of NCs) was administered in all the cases. At 1 h post-administration, the thoracic cavities of the rats were exposed and a microinfusor was deeply inserted into the left ventricle. The right atrium was cut to allow the movement of the administered fluids. Firstly, 100 mL of a solution of 0.1% rhodamine in PBS (pH = 7.4) was introduced through a peristaltic pump (Spetec GmbH, Perimax 12, Erding, Germany) at a flow rate of 20 mL/min. Then, the lungs were fixed employing 50 mL of fixing solution (50 mL of PBS containing 0.1% rhodamine, 0.6% formaldehyde and 0.9% glutaraldehyde, pH = 7.4) at a flow rate of 15 mL/min. After, the lungs were removed and fixed externally for 48 h in 100 mL of 10% buffered neutral formalin. Next, the upper right lobe was sectioned in cuts (50 and 60 µm thick) because it was expected to have the highest amount of formulation [13]. The cuts were placed over a holder and covered with a glass coverslip by an aqueous mounting medium (Fluoromount^®^) to be observed by CLSM. The photographs were taken using a microscope Leica TCS-SP5X-AOBS with a white laser (470–670 nm) and a UV laser (Leica) with LAS AF (Leica Application Suite Advanced Fluorescence) software, using a 63X objective (oil PL APO63x/N.A.1.4-0.6 CS). Data were collected employing sequential mode using green channel (Cu^6^ emission, λ = 455–461 nm) and red channel (rhodamine emission, λ = 553–627 nm), and were superimposed to get a multi-colored image.

#### 2.11.2. In Vivo Gene Expression Study

pCMV-βGal-CS NCs-loaded Ma MS and pCMV-βGal-HA/CS NCs-loaded Ma MS, as well as the controls (CS NCs-loaded Ma MS, HA/CS NCs-loaded Ma MS and pCMV-βGal-Ma MS) were intratracheally administered to anesthetized rats following the method described above. After administration, the cannula was removed from the trachea and the rats’ necks were surgically sutured employing a surgical suture. At 1 or 2 h post-administration, the rats were allowed to recover from anesthesia. Three days post-administration, the rats were sacrificed and their lungs were removed to study the β-galactosidase expression in lung sections using a method previously described [58] that was adjusted in this study. Briefly, the endogenous β-galactosidase activity was inhibited by incubation of the lung tissues in solutions of pH around 7.4. For that, lung slices were incubated in a solution of 1X PBS for approximately 1 h at room temperature. Next, they were fixed in a 1X solution of 2% formaldehyde and 0.2% glutaraldehyde for 30 min at 4 °C and after, they were incubated again in a 1X PBS solution for 1 h at room temperature. The expression of the encoded β-Gal was studied by the 5-Bromo-4-chloro-3-indolylbeta-D-galactopyranoside (X-Gal) reaction. For that, lung slices were incubated in the X-Gal solution as specified by the β-galactosidase reporter gene staining kit. The cuts of lung tissue were removed from the X-Gal solution at 2 h and were washed in MilliQ water during 10 min at room temperature. Then, the lung sections were stained with Fast Red for 5 min and washed in MilliQ water during 1 min at room temperature. Once the lung samples were dried at room temperature, they were observed by light field optical microscopy in a Leica DMRE7 optical microscope (Leica Microsystems Heidelberg GmbH, Germany), using the HC PL APO CS 20x/0.7 lens.

## 3. Results and Discussion

In this study, a new dry powder platform consisting of pDNA-loaded CS-based NCs microencapsulated in Ma MS was developed. The NCs were loaded with a model plasmid (pCMV-βGal) aimed to demonstrate their in vivo transfection potential.

### 3.1. Preparation and Characterization of CS-Based NCs

**Figure 1:**
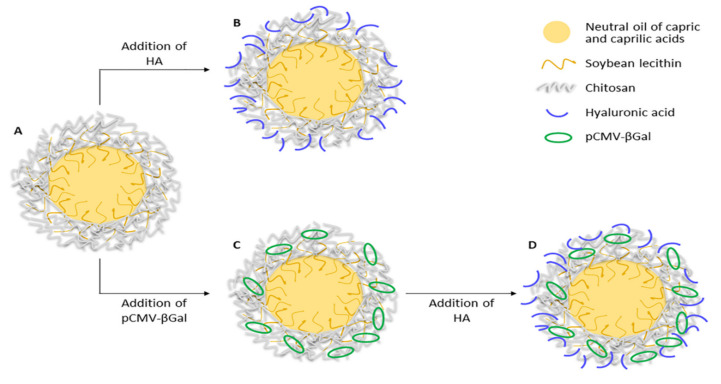
Theoretical structures of: (**A**) CS NC, (**B**) HA/CS NC, (**C**) pCMV-βGal-CS NC and (**D**) pCMV-βGal-HA/CS NC.

Various CS-based NCs formulations (CS NCs, HA/CS NCs, pCMV-βGal-CS NCs and pCMV-βGal-HA/CS NCs) were prepared using the procedure described in the Methodology. Their theoretical structures are shown in Figure 1.

As can be seen in Figure 1, CS NCs present the simplest core-shell structure (Figure 1A). They contain an oily core composed of a mixture of neutral oils of capric and caprilic acids, a soybean lecithin interface and a chitosan coating. The lipophilic core is useful for the encapsulation of hydrophobic molecules. The surfactant lecithin plays an important role in the composition of NCs thanks to its amphiphilic nature. It is found on the contact surface of the two apolar-polar phases (core-shell, respectively), providing binding and stability to the NC structure. The CS coating is hydrophilic and gives positive charge to the surface of the NCs. It allows to load negative molecules, such as HA (Figure 1B), plasmid (Figure 1C) or both (Figure 1D). HA was incorporated on the NCs surface in order to improve the transfection properties. CS-based NCs constitute versatile nanosystems for the simultaneous transport of molecules of different nature, thanks to their structure and composition. We have prepared NCs that simultaneously incorporate plasmid and the antibiotic rifabutin, on the surface and into the core of the NCs, respectively (data not shown).

As shown in Table 2, CS NCs present a particle size of about 160 nm and a positive ζ-potential of approximately +56 mV. The electrostatic association of HA (negatively charged) on their surface caused a slight reduction in size up to 154 nm and a decrease in the ζ-potential to +35 mV, remaining positive. These results could be explained by the electrostatic interaction between the amino groups of CS (positively charged) and the carbonyl groups of the HA (negatively charged), and are consistent with those of NPs prepared with CS, HA and TPP [59,60], in which keeping constant the TPP content, the size and ζ-potential of the NPs decreased as the amounts of HA increased with respect to CS.

When pCMV-βGal was added to the CS NCs, the size of the nanostructures was barely modified (from 160 nm to 165 nm for CS NCs and pCMV-βGal-CS NCs, respectively); while for the HA-containing NCs, slightly increased (from 154 nm to 162 nm for HA/CS NCs and pCMV-βGal-HA/CS NCs, correspondingly) (see Table 2). The ζ-potentials slightly decreased when the plasmid was associated to the NCs, being of approx. +56 mV to +54 mV for unloaded and plasmid-loaded CS NCs, respectively; and of +35 mV to +29 mV for unloaded and plasmid-loaded HA/CS NCs, correspondingly. The decrease of ζ-potential is explained by the electrostatic interaction between the plasmid (negatively charged) and the CS (positively charged). Slight decreases in ζ-potentials were also observed for CS/TPP NPs with increasing amounts of pDNA [18].

In order to determine the in vivo lung distribution of the powders, the CS NCs and HA/CS NCs were labelled with Cu^6^ and were dialyzed previously to their microencapsulation in Ma MS, as indicated in the Methodology section. The labelling of NCs with Cu^6^ hardly affected to their physicochemical properties. Both size and ζ-potential of pre-dialyzed Cu^6^-labelled NCs kept practically the same as those of the unlabeled NCs (see Table 2). The dialysis process did not affect to the size of Cu^6^-labelled NCs but produced a reduction of their ζ-potentials of almost 18 mV and 11 mV for the Cu^6^-CS NCs and Cu^6^-HA/CS NCs, respectively, compared with their pre-dialyzed forms. This could be explained by the elimination of NCs remnants components, probably CS polymeric filaments (positively charged), which were in excess after the NCs formation.

Furthermore, it is very important to notice that all the formulations that contains HA present a decrease of size and of ζ-potential with respect to their counterpart without HA (Table 2). It is due to the electrostatic interactions of the HA to the NCs surface. This binding is more significantly corroborated in the case of the dialyzed NCs. When NCs are dialyzed, the possible debris, impurities and unbound CS and HA are eliminated. Anyway, the dialyzed Cu^6^-HA/CS NCs presented a smallest size and a decrease in ζ-potential with respect to the dialyzed Cu^6^-CS NCs (Table 2), which indicates that HA remained attached to the NCs.

The production yields were around 83% and 79% for the CS NCs and pCMV-βGal-CS NCs, respectively. However, they were lower, but still acceptable, around 67% and 65% for the HA/CS NCs and pCMV-βGal-HA/CS NCs, correspondingly (Table 2). It is striking that the formulations prepared with HA had lower yields with respect to their counterparts without HA, but the same was observed for the CS/HA/TPP NPs [59]: as the proportion of HA increased, the yield of the NPs decreased. This can be explained because it has been experimentally found that it is more difficult to isolate the NCs cream when the formulation contains HA, which results in a lower yield.

**Figure 2:**
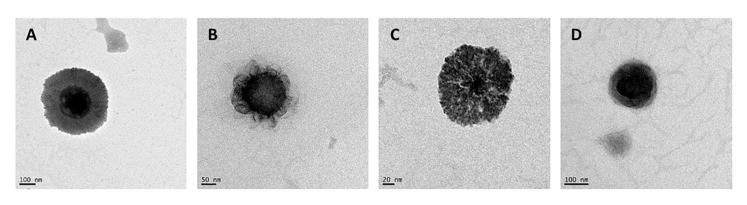
Transmission electron microscopy (TEM) microphotographs of freshly prepared: (**A**) CS NC, (**B**) HA/CS NC, (**C**) pCMV-βGal-CS NC and (**D**) pCMV-βGal-HA/CS NC.

NCs exhibited a spherical morphology with the presence of a core-shell structure as revealed by TEM for the representative formulations (Figure 2). The incorporation of HA produced a change in the appearance of the NCs surface, where HA chains can be appreciated on their surface, generating an external layer (comparison of Figure 2B and Figure 2A). These NCs are in agree with other CS-based NCs previously prepared [61,62,63]. For other hand, the incorporation of pCMV-βGal in the CS NCs modified the appearance of their structure, being the shell more fluted and producing invaginations towards the interior of the core. The presence of lecithin (amphiphilic) allowed the flexibility and deformation of the core-shell interface, maintaining the binding and the stability of the NC structure (Figure 2C). On the other hand, the pCMV-βGal-HA/CS NCs (Figure 2D) adopted a more compact conformation with respect to the HA/CS NCs (Figure 2B). The structures of the representative systems (Figure 2) are consistent with those described in the theoretical scheme (Figure 1), except for the pCMV-βGal-HA/CS NCs, being more compact (Figure 2D).

### 3.2. Determination of the pCMV-βGal Association to the NCs

**Figure 3:**
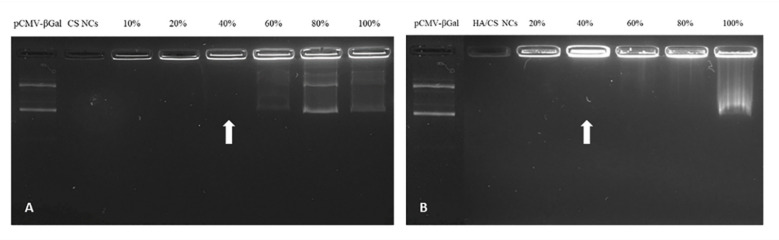
Association of pCMV-βGal to: (**A**) CS NCs and (**B**) HA/CS NCs by agarose gel electrophoresis (1% agarose), at different percentages (up to 100%) with respect to the total amount of CS employed to prepare a batch of NCs.

The ability of the NCs to associate pCMV-βGal was demonstrated by the agarose gel electrophoresis method, using increasing amounts of plasmid up to 100% (*w/w*)—with respect to the total amount of CS used in the preparation of the NCs. As can be seen in the gels shown in Figure 3, corresponding to CS NCs (Figure 3A) and HA/CS NCs (Figure 3B), the free plasmid (in the first well) ran freely, while the control NCs (without plasmid) remained immovable in the second well. However, in presence of both CS NCs and HA/CS NCs, the plasmid stayed bound to the NCs without running from the well (as can be seen in the third and following wells) up to 40% (*w/w*) of plasmid (pointed by arrows in both images of Figure 3A,B). These results reveal a strong plasmid-to-NCs association. With amounts of plasmid higher than 40%, the excess of plasmid ran freely from the wells. Therefore, the decision was made to incorporate pCMV-βGal in the NCs with a theoretical percentage of 40% (*w/w*).

To further characterize the association of the plasmid to the NCs, it was quantified by fluorimetry (using a Qubit^®^) the concentration of free plasmid of the supernatant liquid of the centrifuged plasmid-loaded NCs suspension. The E.E. and D.L. of the NCs (see Table 3) were high and similar for both pCMV-βGal-CS NCs and pCMV-βGal-HA/CS NCs (pCMV-βGal-CS NCs: 91% and 36%, respectively; pCMV-βGal-HA/CS NCs: 89% and 36%, correspondingly). These results are consistent with those of CS/TPP NPs loaded with pDNAs (pEGFP-C1 and pCMVβ-Gal) (70–100%) [18], HA/CS NPs loaded with pDNA (pEGFP-C1) (87–99%) [60] and the HA/CS NPs loaded with siRNA (Luciferase-specific duplex siRNA) (> 95%) [20]. In the last case, although both types of nanostructures (CS-based NCs and CS-based NPs) presented the same CS and HA, the molecular structure of the genetic material was different, being much larger in the case of the plasmid (7164 bp) with respect to siRNA (21 bp), which could influence in the slightly lower E.E. of the CS-based NCs compared to the CS-based NPs.

### 3.3. Preparation and Characterization of the Dry Powders

CS-based NCs were incorporated in Ma MS by spray-drying in form of dry powders to facilitate their pulmonary administration. Spray-drying is a simple and rapid microencapsulation procedure that offers numerous advantages in the development of aerosolizable drug delivery systems. For example, it allows the use of different biomaterials to obtain versatile MS with different structures and surface characteristics—which permit controlling the release of drugs—as well as improve the aerosolization and the in vivo efficacy [64]. It has been reported that the spray-drying temperature do not compromises the stability of the encapsulated thermolabile molecules because, in the solvent evaporation process, the evaporated solvent absorbs a great amount of the heat applied to the system [65].

In this study, Ma was chosen as the spray-drying excipient due to our previous studies that proved the utility of Ma MS as inert carriers for the pulmonary administration of proteins (insulin) and antibiotics (rifabutin and isoniazid) loaded in CS-based NPs, SLN and MOFs [13,51,52,53,54,66]. Furthermore, it is important to mention that we demonstrated the thermoprotective effect of Ma on insulin loaded in HA/CS NPs [48], which justifies its use for the microencapsulation of nanocarriers loaded with thermolabile molecules. Further, in this work, the liquid oily core of the NCs was taken into consideration and the inlet temperature was set in a low value (T_Inlet_ = 105 °C) that, in turn, favored obtaining a low outlet temperature (T_Outlet_) (Table 4). This, together with the thermoprotective effect of Ma [48], guaranteed the physical stability of the NCs, as well as of the associated macromolecule.

The T_Outlets_ (°C) and P.Y. (*w/w*, %) are collected in the Table 4. As expected, the T_Outlets_ were low (between 55 and 60 °C), suitable for the NCs microencapsulation. In addition, the obtained yields oscillated between 64% and 71%, approximately. These values were high and similar to those reported for the spray-drying of CS-based NPs (CS/TPP NPs, CS/HA/TPP NPs, CS/carboxymethyl-β-cyclodextrin/TPP NPs) (65–70%) [13,48,59,67], of more compact structure (without liquid oily core) and whose spray-drying temperatures were as high as 160 and 170 °C. Furthermore, the obtained P.Y. were higher than those resulting from the microencapsulation of nanostructures containing lipids, e.g., lipid/CS NPs complexes (approx. 50%) [53] and SLN made of glyceryl tristearate or glyceryl dibehenate (47% to 60%) [51], which were also prepared using a low spray-drying temperature (103 °C) [51].

**Figure 4:**
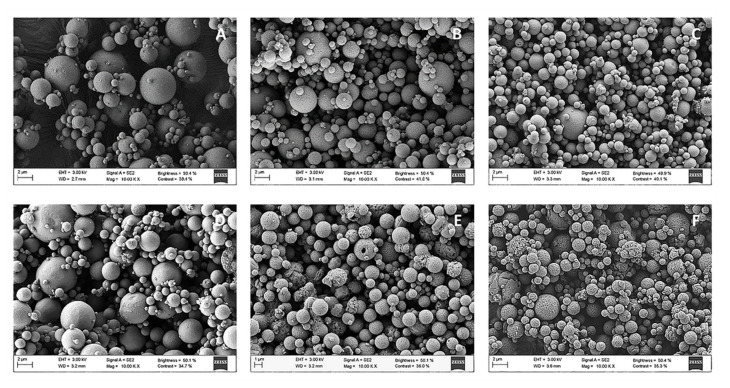
Scanning electron microscopy (SEM) microphotographs of: (**A**) Ma MS, (**B**) CS NCs-loaded Ma MS, (**C**) HA/CS NCs-loaded Ma MS, (**D**) pCMV-βGal-Ma MS, (**E**) pCMV-βGal-CS NCs-loaded Ma MS and (**F**) pCMV-βGal-HA/CS NCs-loaded Ma MS.

SEM microphotographs (Figure 4) show that individual Ma MS were spherical, somewhat heterogeneous in size, with well-defined limits (Figure 4A). When the CS NCs or HA/CS NCs were included in the Ma MS (Figure 4B,C, respectively), it is important to note that the resulting micro-nanostructures—in addition to being spherical and presenting well-defined limits—were comparatively less aggregated and smaller in size than the previous ones. As expected, the inclusion of plasmid in the MS (pCMV-βGal-Ma MS, pCMV-βGal-CS NCs-loaded Ma MS and pCMV-βGal-HA/CS NCs-loaded Ma MS) (Figure 4D–F, correspondingly) did not affect their characteristics.

Dry powders were characterized in terms of geometric diameter (µm), apparent and real densities (g/cm^3^) and theoretical aerodynamic diameter (µm). CS NCs- and HA/CS NCs-loaded Ma MS showed geometric diameters of around 2.10 µm and 2.77 µm, respectively (Table 5), being smaller than that of the control MS (without NCs), whose geometric diameter was of 3.75 µm. The incorporation of pCMV-βGal in the MS (pCMV-βGal-Ma MS, pCMV-βGal-CS NCs-loaded Ma MS and pCMV-βGal-HA/CS NCs-loaded Ma MS) hardly affected their geometric diameters. The apparent density of the Ma MS (approx. 0.50 g/cm^3^) was higher than those of the NCs-loaded Ma MS (CS NCs-loaded Ma MS: 0.44 g/cm^3^ and HA/CS NCs-loaded Ma MS: 0.42 g/cm^3^), but their real density was lower (Ma MS: 1.29 g/cm^3^ and NCs-loaded Ma MS: 1.43 g/cm^3^). The higher geometric diameter and lower real density of Ma MS compared to the NCs-loaded Ma MS, suggests that the structure of the Ma MS is less compact and more porous than those of the NCs-loaded Ma MS.

The Ma MS showed a higher theoretical aerodynamic diameter (4.21 µm) than the NCs-loaded Ma MS (CS NCs-loaded Ma MS: 2.51 µm and HA/CS NCs-loaded Ma MS: 3.35 µm). This was expected taking into account the direct relationship between the D_g_ and the theoretical D_aer_ (see formula in Section 2.9.). To achieve an adequate pulmonary delivery, the MS must have approximately an aerodynamic diameter between 1–5 µm [39,68,69,70]. If they are smaller than 1 µm, the particles will be expelled with the air, but if they are greater than 5 µm, they will remain in the upper respiratory tract [66,69]. In our previous studies, Ma MS containing CS/TPP NPs [13,59,67,71] and SLN [51] showed values of geometric and aerodynamic diameters between 2–5 µm, apparent densities of 0.3–0.6 g/cm^3^ and real densities of 1.3–1.5 g/cm^3^, suitable to achieve the deep lung. In this work, the theoretical aerodynamic diameters of the dry powders were also within the optimal range (1–5 µm). On other hand, the apparent densities were low, which is generally associated with good aerodynamic flow behavior [72,73]. Therefore, the micro-nanoplatforms designed and prepared in this work are theoretically suitable for their administration to the deep lung (bronchioles and alveoli) [74].

### 3.4. Release of NCs from Dry Powders

**Figure 5:**
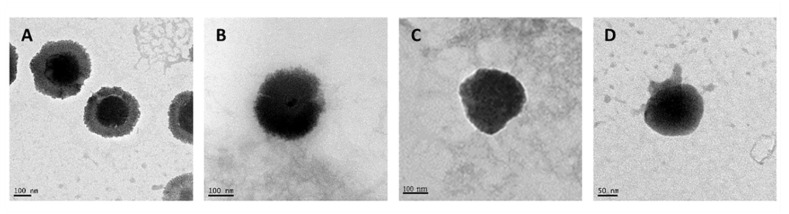
TEM microphotographs of: (**A**) CS NCs, (**B**) HA/CS NC, (**C**) pCMV-βGal-CS NC and (**D**) pCMV-βGal-HA/CS NC released from Ma MS in MilliQ water.

To check the in vitro capacity of the MS to release NCs, the dry powders were incubated in MilliQ water and in simulated pulmonary medium. This is a mixture of the lung surfactant Curosurf^®^ and PBS (0.1% Curosurf^®^ in 10 mM PBS (*v/v*)) with a pH of 7.4—like the pH of the pulmonary fluid, plasma and interstitial fluid [75]. It was observed that in both media, the Ma rapidly dissolved releasing the NCs. As can be seen in the TEM microphotographs, the released NCs (Figure 5) were morphologically similar to the freshly prepared NCs (Figure 2), demonstrating that the spray-drying process did not affect to the structure of the NCs, as expected [65]. Therefore, it is predictable that NCs will be released also unchanged in the lung fluid.

**Figure 6:**
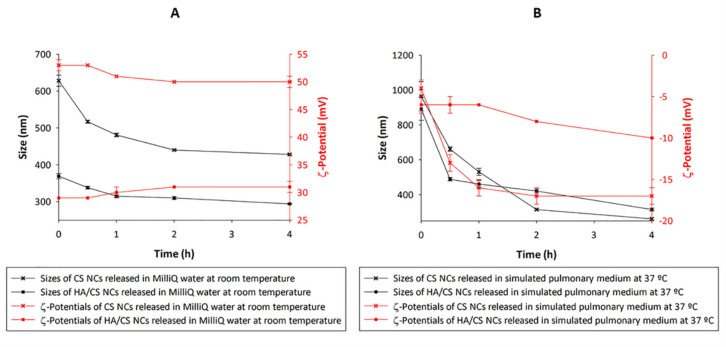
Sizes and ζ-potentials of CS-based NCs released from Ma MS in: (**A**) MilliQ water at room temperature and (**B**) simulated pulmonary medium at 37 °C, at different times (0, 0.5, 1, 2 and 4 h) (mean ± S.D.; *n* = 3).

Figure 6 shows the sizes and ζ-potentials of the CS NCs and the HA/CS NCs released from Ma MS in MilliQ water (Figure 6A) and in simulated pulmonary medium (Figure 6B) during 4 h. Following the incubation of the MS in both aqueous media, the NCs showed an increase of size with respect to the fresh NCs, probably due to the presence of Ma remaining of the MS—still in process of release of the NCs and other loose debris of Ma. The increase of size of the NCs released in simulated pulmonary medium was greater than in MilliQ water, surely caused by the electrostatic attraction of negatively charged molecules from the medium to the positive surface of the NCs—fact that has been reported for other nanosystems microencapsulated in Ma [66]—but it is not relevant. As time progressed, the NCs sizes decreased maintaining themselves above of the fresh NCs sizes, but still within the nanometric range—suitable for the purpose of this study. When CS NCs and HA/CS NCs were released in MilliQ water, the ζ-potentials did not vary, but in simulated pulmonary medium the NCs acquired negative ζ-potentials—probably due to the presence of negative molecules of the medium joined by electrostatic attractions to the positive surface of the NCs.

Count rates are shown in Table 6. For fresh CS NCs, the mean was of approx. 273.8 ± 7.3 kcps. The count rate ranges of CS NCs released of 0 to 4h varied from 262 to 359 kcps and from 280 to 399 kcps when they were released in MilliQ water and in simulated pulmonary medium, respectively. The mean count rate for fresh HA/CS NCs was approx. 198 ± 1.9 kcps and for the released ones (of 0 to 4h), the ranges varied from 102 to 216 kcps and from 185 to 226 kcps in MilliQ water and in simulated pulmonary medium, correspondingly. This indicates that the CS-based NCs were released in great quantities from the Ma MS. Furthermore, at the beginning of the NCs release, a lower count rate was observed compared to that seen at 4 h because the release process was beginning—which is also confirmed by the larger size of the particles at that initial time (Figure 6). This increase in size, as well as a lower number of particles at the beginning of the release, can be explained by the presence of Ma, which was still releasing NCs. Therefore, as time passed and the Ma dissolved, the mean count rate increased as expected, while a decrease in the particle size occurred.

Taking into account the high solubility of Ma in the investigated aqueous media and the results obtained in this study, it is expected that the dry powders administrated in vivo release the NCs in the lung fluid.

### 3.5. In Vivo Studies

#### 3.5.1. Lung Distribution of Microencapsulated NCs

**Figure 7:**
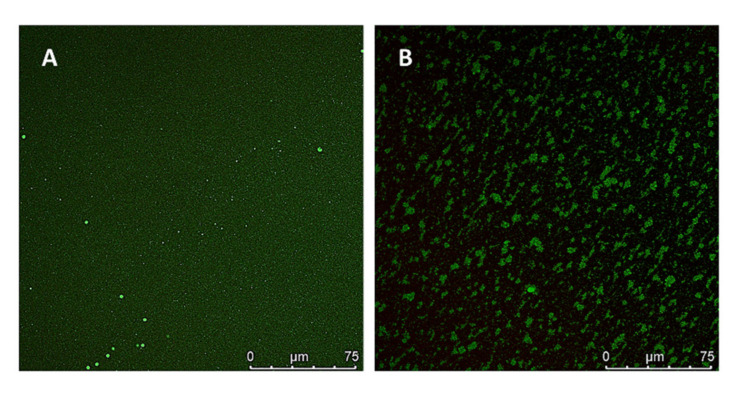
Confocal laser scanning microscopy (CLSM) microphotographs of: (**A**) pre-dialyzed Cu^6^-CS NCs and (**B**) dialyzed Cu^6^-CS NCs.

The in vivo pulmonary distribution of microencapsulated NCs was investigated using Cu^6^-labelled NCs and the CLSM technique, as described in Section 2.11.1. The Cu^6^-NCs were dialyzed prior to their microencapsulation to remove the excess of free Cu^6^. In addition, the Cu^6^-CS NCs were used to verify that the encapsulated Cu^6^ was not released from the nanostructures during the study. Indeed, the fluorescent green signal of the non-encapsulated Cu^6^ is distinguished outside the Cu^6^-CS NCs in the microphotograph of the pre-dialyzed Cu^6^-CS NCs (Figure 7A); but not in the microphotograph of the Cu^6^-CS NCs after the dialysis (Figure 7B). Furthermore, the dialyzed Cu^6^-CS NCs appears joint together in groups, unlike the pre-dialyzed Cu^6^-CS NCs that are isolated. This can be explained by the different distribution adopted by both Cu^6^-CS NCs samples between the slide and the cover during the experiment. In the case of the pre-dialyzed Cu^6^-CS NCs, the presence of an excess of Cu^6^ between the nanostructures may keep them mostly isolated from each other, probably due to the apolar nature of the Cu^6^. In this respect, it has been verified that both the labelling of NCs with Cu^6^ and the dialysis process did not lead to the aggregation of NCs due to that the sizes of the pre- and dialyzed Cu^6^-NCs were practically the same as the unlabeled NCs (see results in Table 2).

**Figure 8:**
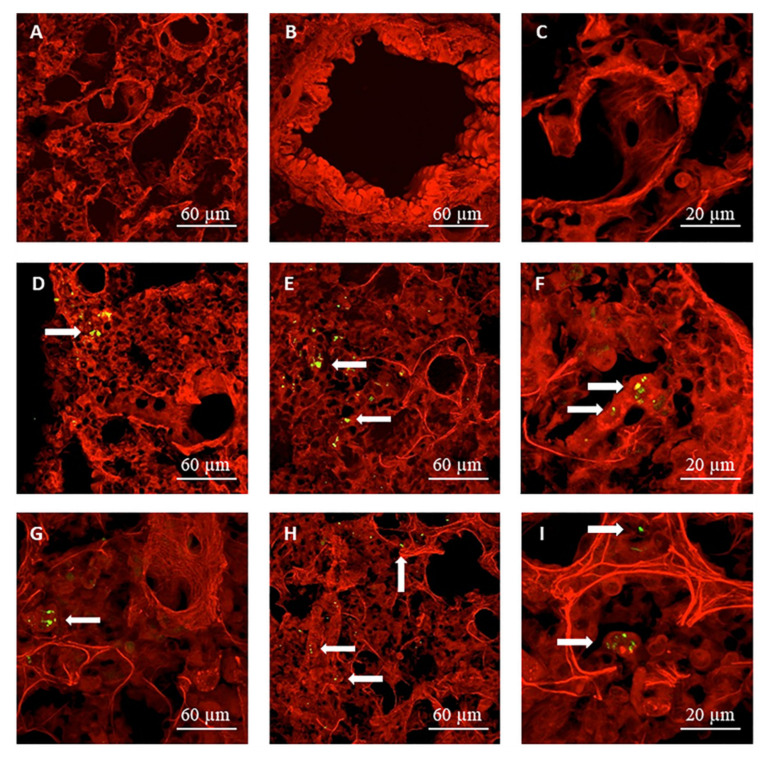
CLSM microphotographs of alveoli: (**A–C**) control (without powder), (**D–F**) at 1 h post-administration of Cu^6^-CS NCs-loaded Ma MS and (**G–I**) at 1 h post-administration of Cu^6^-HA/CS NCs-loaded Ma MS.

Representative CLSM images of lung sections obtained following the administration of the Cu^6^-labelled micro-nanostructured formulations to rats, are shown in Figure 8, in which the fluorescent green signal associated to the NCs is visible. The images in Figure 8A–C correspond to the control lung tissue (without powder), while those in Figure 8D–F and Figure 8G–I represent lung tissues of rats that were treated with Cu^6^-CS NCs-loaded Ma MS and Cu^6^-HA/CS NCs-loaded Ma MS, respectively. At 1h post- administration of both formulations, micro-nanostructures were detected in the lung epithelium (indicated by arrows in Figure 8), being some of them captured by macrophages (Figure 8F,I). Macrophage phagocytosis depend on several factors, including the nature and charge of the particles, size and shape, concentration and contact time, between others [13]. In this sense, macrophages have preference for hydrophobic particles, or with a high surface charge (either positive or negative), with a size between 1–6 µm, spherical and non-porous [76]. For that, we cannot exclude that some NCs still included in the MS were phagocytosed. It must be taking into account that—although it was demonstrated in vitro that NCs were rapidly released from MS due to the fast dissolution of the Ma in the aqueous media (Figure 6A,B)—it is reasonable to think that the in vivo release of the NCs is slower due to the small volume of alveolar fluid [13]. The fact that the micro-nanostructures were distributed in the deep lung, as well as that some MS were phagocytosed, was also observed with the CS/TPP-insulin NPs-loaded Ma MS aimed at protein lung absorption [13]. These results confirm that the new micro-nanostructured platform has a great potential for the pulmonary administration of nanostructures, more specifically of NCs, leading to their release in the deep lung. Next study was aimed to demonstrate the ability of the plasmid-loaded micro-nanostructured dry powders to produce lung transfection after their in vivo administration.

#### 3.5.2. In Vivo Gene Expression Study

**Figure 9:**
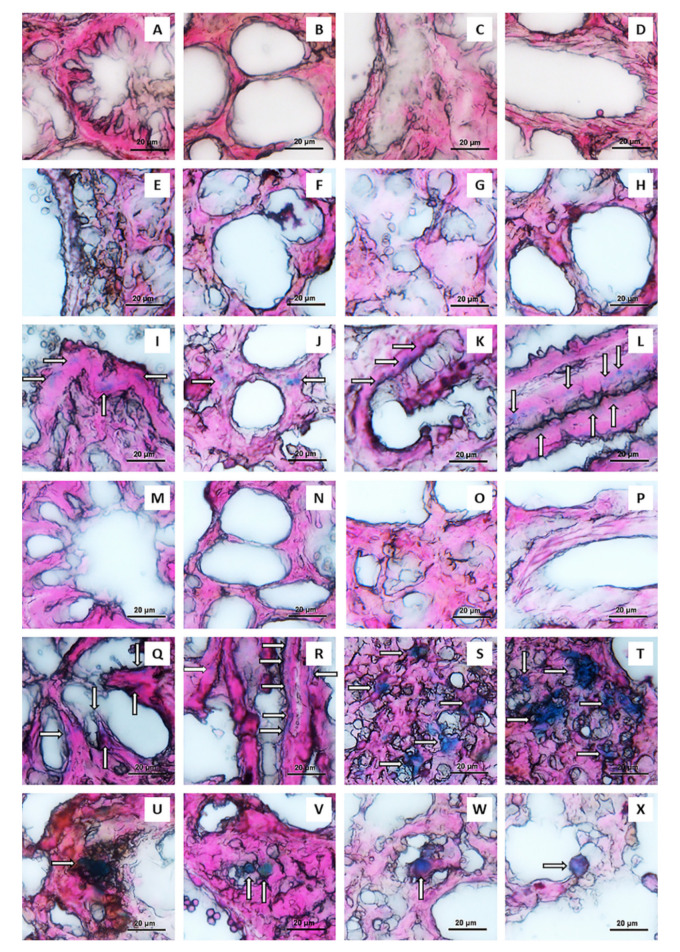
Light field optical microscopy images of rat lung: (**A–D**) without powder administration, and post-administration of: (**E–H**) CS NCs-loaded Ma MS, (**I–L**) pCMV-βGal-CS NCs-loaded Ma MS, (**M–P**) HA/CS NCs-loaded Ma MS, (**Q–T**) pCMV-βGal-HA/CS NCs-loaded Ma MS and (**U–X**) pCMV-βGal-Ma MS.

The plasmid pCMV-βGal (or LacZ gene) used in this study is one of the most used reporter genes for the evaluation of gene transfection efficiency. It codes the enzyme β-Gal. The substrate used to test the functionality of β-Gal is 5-Bromo-4-chloro-3-indolyl-beta-D-galactopyranoside (X-Gal). When lung tissues of rats treated with plasmid-loaded formulations were incubated within X-Gal solution, the β-Gal present in the tissues transforms the X-Gal molecules in blue precipitates. It is important to point out that the tissues of mammals also possess endogenous enzymes with the same β-galactosidase activity. Therefore, it was crucial to inhibit the endogenous β-galactosidase activity to avoid false positives. To do that, the tissues were previously processed properly to inhibit these enzymes (by incubation in solutions of pH around 7.4, as previously explained in Section 2.11.2.) [58,77]. In order to inhibit the endogenous β-galactosidase activity [58,77], it was also considered that the pH at which the exogenous β-galactosidase activity is highest is above than the pH at which the mammalian β-galactosidase activity is greatest. Therefore, this study was made at a pH of 7.4 to ensure the inhibition of the mammalian β-galactosidase activity and to maintain optimal the exogenous β-galactosidase activity. In addition, it was necessary to find a balance in the incubation time of the lung tissues in the X-Gal solution. This inhibition could be maintained until the 2 h, once the reaction with X-Gal began. Therefore, the lung tissues were incubated for exactly 2 h in the X-Gal solution. Furthermore, it was better to process the lung in sections than in block because it allows a more effective inhibition of the endogenous β-galactosidase activity, as well as a better detection of the exogenous β-galactosidase activity [58]. Therefore, following the pulmonary administration to rats of pCMV-βGal-CS NCs-loaded Ma MS and pCMV-βGal-HA/CS NCs-loaded Ma MS, as well as their controls (CS NCs-loaded Ma MS, HA/CS NCs-loaded Ma MS and pCMV-βGal-Ma MS), the β-Gal expression was studied in sections of the lungs extracted three days after the administration of the powders [18]. As can be seen in the light field optical microscopy images of the Figure 9, sections of rat lungs that were not treated with powders (Figure 9A–D) show no blue deposits, indicating that the endogenous β-galactosidase activity was correctly inhibited to avoid false positives. Neither appeared blue deposits when the rats were administered with the control powders without plasmid: CS NCs-loaded Ma MS (Figure 9E–H) and HA/CS NCs-loaded Ma MS (Figure 9M–P). On the contrary, blue deposits (indicated with arrows) clearly and reproducibly appeared in the lungs of rats treated with the plasmid-loaded formulations (pCMV-βGal-CS NCs-loaded Ma MS: Figure 9I–L; pCMV-βGal-HA/CS NCs-loaded Ma MS: Figure 9Q–T; pCMV-βGal-Ma MS: Figure 9U–X), indicating efficient in vivo gene expression.

Blue deposits were evidenced as result of the enzymatic reaction of β-Gal—which was formed thanks to the expression of the intact structure of pCMV-βGal in the lung tissue. Specifically, important differences were observed between the transfection patterns of the plasmid-loaded formulations, depending on whether or not the genetic material was associated to NCs and whether or not the NCs contained HA. Indeed, in the lungs of rats treated with dry powders of Ma MS containing naked plasmid (pCMV-βGal-Ma MS), the blue deposits formed clusters. This might be explained because the MS, in contact with the lung fluid, released the plasmid very rapidly in the lung epithelium (Figure 9U–X). In contrast, when pCMV-βGal was previously incorporated to CS NCs and HA/CS NCs (pCMV-βGal-CS NCs-loaded Ma MS: Figure 9I–L; pCMV-βGal-HA/CS NCs-loaded Ma MS: Figure 9Q–T), the resulting blue deposits were more scattered throughout the lung epithelium. Furthermore, as expected [20,25,26,27,78], the addition of HA to the coat of the CS NCs improved their transfection properties. As can be seen in Figure 9S,T, for pCMV-βGal-HA/CS NCs-loaded Ma MS, there were blue deposits extended beyond the edge of the pulmonary alveoli and the transfection occurred homogeneously over a wider area. This difference on the transfection patterns demonstrates that HA was attached to the NCs surface—which corroborates the results of the Table 2 and Figure 2B. HA promotes the internalization of nanostructures by increasing both specific and non-specific interactions with cells [27]. During internalization, HA/CS NCs are also expected to enter into non-lysosomal vesicular compartments, accumulating in the perinuclear area and in the cell nucleus [79]. Furthermore, HA promotes the gene expression by acting as a transcriptional activator, probably by loosening the tight bond between the nanosystem and the gene [78]. This improvement in the transfection efficiency was also observed for CS/HA/TPP NPs prepared using pEGFP, pβ-gal or siRNA [20,27]. In these studies, it was demonstrated that the transfection capacity was strongly related to the composition, increasing the level of gene expression as the amount of HA in the NPs was higher.

Dry powders are habitually more preferred for inhalation than their equivalents in liquid formulations due to their better stability [13]. It has been reported the administration of naked pDNA inhaled in form of dry powders prepared with different excipients, resulting in a better transfection than their counterparts in suspension. In that study, the level of transfection was highly dependent on the excipient used for the preparation of the powders, being the MS prepared with HA the ones that gave the best results [80]—which could be expected taking into account the HA transfection enhancer effect. CS NCs have already been proposed for lung delivery of genes [37], but they had not been assessed in vivo. The main novelty of this work is that we devised the microencapsulation of the genetic material-loaded CS-based NCs in Ma MS to facilitate their pulmonary administration in dry powders form, leading to revealing results. Furthermore, it is very important to highlight that the systems presented in this work are not toxic for the A549 cell line (adenocarcinoma human alveolar basal epithelial cells) (results not shown). Correspondingly, the results of the histopathological examination of the lung sections (Figure 9) are remarkable, taking into account that the lungs were removed 3 days after the administration of the dry powder formulations, sufficient time to evaluate the possible toxicity of the micro-nanosystems. As can be seen in the Figure 9, the absence of both pulmonary embolism and inflammation was observed, as well as the presence of normal parenchyma, with alveoli of thin wall. These results are comparable to those observed in lung tissue sections not treated with formulations (Figure 9A–D), which visibly confirm the non-toxicity and, hence, the security of the formulations for pulmonary administration. In addition, an interesting advantage of the CS-based NCs is that they can be used for a combined gene therapy, loading the genetic material on the coat and an adjuvant lipophilic molecule, in the core. This molecule can enhance the transfection, like capsaicin [37]—which decreases the thickness of the mucus layer [81] and opens the intercellular tight junctions in a reversible way [82,83]. The versatility of the simultaneous transport of HA and different active molecules in the same simple structure allows to carry out a synergistic transfection effect, being of enormous value for a more effective treatment of genetic lung diseases. Taking advantage of the versatility of the NCs, we have in mind the idea of incorporating therapeutic genetic material (on the surface of NCs) and an antibiotic in the same nanostructure, in order to combine gene and anti-infective therapy for the treatment of certain pulmonary infectious pathologies.

## 4. Conclusions

The developed pCMV-βGal-CS-based NCs showed particle sizes in the nano-range, positive ζ-potentials and adequate plasmid payloads. They were successfully microencapsulated in Ma MS by spray-drying, resulting in dry powders of suitable characteristics for lung administration. The Ma MS act as an inert pulmonary carrier of the plasmid-loaded NCs, releasing them rapidly in aqueous media. The released NCs keep suitable physicochemical characteristics. The results of the in vivo studies revealed that this platform is capable of reaching the deepest lung region and producing transfection in the lung tissue, as it was demonstrated by the X-Gal reaction. This highlights the important conclusion that the CS-based NCs are able to release the plasmid maintaining its structural and functional characteristics. This transfection is more relevant for the HA/CS NCs, which resulted in a greater transfection efficiency, in a more extended area, of a more homogeneous way in the lung tissue. The final conclusion is that the microencapsulated CS-based NCs constitute a promising platform for the delivery of genes to the lung and have a great potential for the treatment of genetic lung diseases.

## Figures and Tables

**Table 1:** Theoretical concentrations and volumes of the components employed to prepare pCMV-βGal-loaded chitosan (CS)-based nanocapsules (NCs).

Formulation	MilliQ Water (µL)	pCMV-βGal (628 µg/mL) (µL)	CS NCs Suspension (34.3 mg/mL) (µL)	Hyaluronic Acid (HA) Solution (625 µg/mL) (µL)
pCMV-βGal-CS NCs	125	125	250	-
pCMV-βGal-HA/CS NCs	125	93.80	250	31.20

**Table 2:** Physicochemical properties and production yields of CS-based NCs (-Coumarin 6, Cu^6^) (mean ± S.D.; *n* = 3).

Formulation	Size Range (nm)	PdI	ζ-Potential Range (mV)	Production Yield (%)
CS NCs	160 ± 3	0.19	+56.5 ± 1.4	83 ± 4
HA/CS NCs	154 ± 2	0.15	+34.7 ± 0.8	67 ± 7
pCMV-βGal-CS NCs	165 ± 4	0.25	+54.0 ± 1.6	79 ± 6
pCMV-βGal-HA/CS NCs	162 ± 3	0.21	+29.2 ± 1.2	65 ± 8
Pre-dialyzed Cu^6^-CS NCs	161 ± 2	0.21	+57.4 ± 0.6	-
Pre-dialyzed Cu^6^-HA/CS NCs	155 ± 1	0.16	+37.5 ± 0.4	-
Dialyzed Cu^6^-CS NCs	162 ± 1	0.20	+39.6 ± 0.1	-
Dialyzed Cu^6^-HA/CS NCs	156 ± 2	0.15	+26.2 ± 0.1	-

**Table 3:** Encapsulation efficiency (E.E.) and drug loading (D.L.) of pCMV-βGal to the CS-based NCs (mean ± S.D.; *n* = 3).

Formulation	E.E. (%)	D.L. (%)
pCMV-βGal-CS NCs	90.6 ± 0.9	36.2 ± 0.4
pCMV-βGal-HA/CS NCs	89.0 ± 4.8	35.6 ± 1.9

**Table 4:** Outlet temperatures (T_Outlets_) and process yields (P.Y.) of the powder samples obtained by spray-drying (feed rate: 5 mL/min, aspirator: 100%, nozzle diameter: 0.7 mm, nozzle cleaner: 5, inlet temperature (T_Inlet_): 105 ± 2 °C and air flow rate: 600 Nl/h) (mean ± S.D.; *n* = 3).

Dry Powder	T_Outlet_ (°C)	P.Y. (*w/w*, %)
Mannitol (Ma) microspheres (MS)	56	64 ± 3
CS NCs-loaded Ma MS	60	67 ± 5
HA/CS NCs-loaded Ma MS	58	67 ± 6
pCMV-βGal-Ma MS	57	70 ± 5
pCMV-βGal-CS NCs-loaded Ma MS	55	69 ± 8
pCMV-βGal-HA/CS NCs-loaded Ma MS	58	71 ± 8

**Table 5:** Physical and aerodynamic properties of the powder samples obtained by spray-drying (feed rate: 5 mL/min, aspirator: 100%, nozzle diameter: 0.7 mm, nozzle cleaner: 5, T_Inlet_: 105 ± 2 °C and air flow rate: 600 Nl/h) (mean ± S.D.; *n* = 3).

Dry Powder Samples	Geometric Diameter (µm)	Apparent Density (g/cm^3^)	Real Density (g/cm^3^)	Theoretical Aerodynamic Diameter (µm)
Ma MS	3.75 ± 1.56	0.50 ± 0.01	1.29 ± 0.01	4.21 ± 0.01
pCMV-βGal-Ma MS	2.85 ± 1.49	-	-	-
CS NCs-loaded Ma MS	2.10 ± 0.86	0.44 ± 0.02	1.43 ± 0.01	2.51 ± 0.02
pCMV-βGal-CS NCs-loaded Ma MS	1.98 ± 0.70	-	-	-
HA/CS NCs-loaded Ma MS	2.77 ± 1.35	0.42 ± 0.02	1.43 ± 0.02	3.35 ± 0.02
pCMV-βGal-HA/CS NCs-loaded Ma MS	2.43 ± 1.32	-	-	-

**Table 6:** Count rate ranges (kcps) of fresh NCs and NCs released in MilliQ water at room temperature and in simulated pulmonary medium at 37 °C for 4 h (*n* = 3).

Types of CS-Based NCs	Count Rate (kcps)
CS NCs	266–281
CS NCs released in MilliQ water	262–359
CS NCs released in simulated pulmonary medium	280–399
HA/CS NCs	196–200
HA/CS NCs released in MilliQ water	102–216
HA/CS NCs released in simulated pulmonary medium	185–226

## Data Availability

Not applicable.
